# SBA-15 Type Mesoporous Silica Modified with Vanadium as a Catalyst for Oxidative and Extractive Oxidative Desulfurization Processes

**DOI:** 10.3390/ma17164041

**Published:** 2024-08-14

**Authors:** Ardian Nurwita, Katarzyna Stawicka, Maciej Trejda

**Affiliations:** Department of Heterogeneous Catalysis, Faculty of Chemistry, Adam Mickiewicz University in Poznań, Uniwesytetu Poznańskiego 8, 61-614 Poznań, Poland; ardnur@amu.edu.pl (A.N.); katarzyna.stawicka@amu.edu.pl (K.S.)

**Keywords:** desulfurization, dibenzothiophene, mesoporous silica, vanadium

## Abstract

One of the current challenges is the reduction of sulfur emitted into the atmosphere, usually in the form of sulfur oxides generated by fossil fuel combustion. To achieve this goal, the sulfur content should be reduced in fuel. In this context, vanadium-containing materials based on SBA-15 mesoporous silica supports and two different sources of vanadium were prepared, characterized, and applied as catalysts for oxidative desulfurization (CODS) and extractive oxidative desulfurization processes (ECODSs). The novelty of this work was the comparative study of vanadium-containing materials in two desulfurization systems. The properties of the catalysts, the concentration and state of vanadium species, and their role in the catalytic process were examined by low-temperature nitrogen physisorption, XRD, UV-Vis, XPS, and chemisorption of pyridine combined with FTIR spectroscopy. The catalytic performance of the material prepared using ammonium metavanadate was superior to that of the catalyst obtained using vanadium(IV) oxide sulfate, which was explained by a higher concentration of vanadium species on the surface of the support and their lower oxidation state in the former. Both types of catalysts showed high activity and stability in the ECODS process.

## 1. Introduction

The important role of catalysis in modern society is undisputable. It is estimated that ca. 90% of industrial chemical processes are carried out by using at least one catalyst [1]. Moreover, ca. 20% of the world economy directly or indirectly depends on catalysis. In this light, the continuous interest in research related to catalytic processes is not surprising. One of the directions of studies in the area is the heterogenization of processes aimed at designing and applying new solid catalytic materials. One of the benefits of the usage of solid catalysts for processes carried out in the liquid phase is the ease of their separation from the reaction mixture, which reduces the cost of product purification. For the processes that require the presence of acid or base catalysts, another important benefit of using solid catalysts is the lack of corrosion [2,3,4]. However, there are some risks that need to be considered. One of them is the potential leaching of the active phase to the reaction medium. To overcome this threat, the application of proper support for the active phase should be taken into account [5,6].

One of the very common supports applied for catalyst preparation is silica. For instance, it has been used for the large-scale production of sulfuric acid, as a support for vanadium pentoxide. The end of the last century brought a new class of ordered silicas, which are mesoporous in nature, like MCM-41 or SBA-15 [7,8]. MCM-41 material (an acronym for Mobile Composition of Matter), which belongs to the M41S family, consists of hexagonal channels with diameters usually between 2 and 10 nm and has a relatively large specific surface area, usually ca. 1000 m^2^/g. MCM-41 silica is synthesized at a high pH value close to 11. SBA-15 (an acronym for Santa Barbara Amorphous) is quite similar to MCM-41; however, it has thicker walls, which makes this material more stable for hydrothermal treatment. Moreover, the synthesis condition of SBA-15 differs from MCM-41. SBA-15 is synthesized in acidic media; thus, the pH of the synthesis mixture is low. Moreover, because of the application of Pluronic P123 as a template during the synthesis of SBA-15, the resulting samples also show some microporosity, located in the material walls. This is a result of the occluding of the template in the wall of SBA-15 during the synthesis. Furthermore, the template is removed during calcinations, leaving the system of micropores. Materials with pores that are regular in size offer a space for reagents but also for active sites of catalysts. The incorporation of active sites exclusively in the pores of ordered porous materials may give additional benefits, such as the shape-selectivity of the catalytic processes in which they are applied.

An important challenge of chemical research is reducing the amount of sulfur emitted into the atmosphere, usually in the form of sulfur oxides generated in the process of fossil fuel combustion. One way to achieve this goal is to decrease the sulfur content in fuels. Nowadays, this is realized by the hydrodesulfurization process (HDS) of crude oil. This process requires high temperatures (300–450 °C) and pressure in the range of 20–100 atm. Moreover, hydrogen is used as a reactant. It is accompanied by side processes such as the hydrogenation of unsaturated bonds of hydrocarbons present in crude oil. In addition, this process is insufficient for the reduction of organic polyaromatic compounds containing sulfur. However, the latter compounds can be quite easily removed by catalytic oxidative desulfurization (CODS) using hydrogen peroxide as an oxidant, followed by an extraction process. The CODS process can be carried out in much milder reaction conditions [9], which is important from an economical point of view. Until now, different materials have been tested as catalysts of the CODS process, including those containing -SO_3_H active centers and Brønsted acid sites [10,11], as well as those containing Lewis acid sites, like titanium [12,13], tungsten [6,14], molybdenum [15,16,17], and vanadium [18,19,20].

The aim of our study was to investigate the effects of the combination of the above-described advantages of SBA-15 mesoporous silica with the ability of vanadium species, used as active centers, to interact with hydrogen peroxide-generating reactive oxygen species. The novelty of this work is the comparative study of vanadium-containing materials in two desulfurization systems. Thus, the performance of the obtained materials was tested in catalytic oxidative desulfurization and also combined with simultaneous extraction. As the efficiency of catalytic reactions depends on the type of active centers, two types of vanadium of different origins were tested, i.e., ammonium metavanadate (NH_4_VO_3_) and vanadium(IV) oxide sulfate (VOSO_4_).

## 2. Materials and Methods

### 2.1. Materials

Tetraethyl orthosilicate (TEOS) (>99%), Pluronic P123, ammonium metavanadate (>99%), vanadium(IV) oxide sulfate (>98%), dibenzothiophene (98%), dodecane, pyridine (>99%), and hydrogen peroxide (30%) were purchased from Sigma-Aldrich (St. Louis, MO, USA). HCl (35%) was purchased from Stanlab (Lublin, Poland). Acetonitrile (>99%) was purchased from EUROCHEM BGD (Tarnów, Poland).

### 2.2. Preparation of SBA-15 Support

The ordered mesoporous silica, SBA-15, was obtained via hydrothermal synthesis [21]. At first, Pluronic P123 (Poly(ethylene glycol)-block-poly(propylene glycol)-block poly(ethylene glycol)) (4 g), applied herein as a nonionic surfactant, was dissolved in 150 mL of a 0.7 M solution of HCl at 40 °C in a polypropylene bottle. To this mixture, TEOS (8.527 g), as a silica source, was added dropwise upon continuous stirring. Finally, the mixture was stirred at 40 °C for 20 h and then kept at 100 °C under steady state conditions in an oven for the next 24 h. Finally, the white solid product was filtered off, washed with deionized water (1200 mL), and dried at room temperature. The template was removed by calcination in an oven at 500 °C for 6 h with a temperature ramp of 5 °C/min.

### 2.3. Modification of SBA-15 with Vanadium Species

Vanadium species were incorporated on the SBA-15 silica surface via incipient wetness impregnation. Prior to modification, 1.0 g of SBA-15 support was outgassed in an evaporator flask for 1 h at 100 °C. Then, the mesoporous silica was filled with an aqueous solution of vanadium precursors, i.e., ammonium metavanadate (NH_4_VO_3_) or vanadium(IV) oxide sulfate (VOSO_4_). The amount of vanadium in the solution was sufficient to obtain 2 wt.% of this metal in the final samples. The obtained mixture was stirred and heated in an evaporator flask at 80 °C for 1 h. The powder obtained was dried at 110 °C for 18 h and calcined at 500 °C for 6 h in the air under steady-state conditions at a heating rate of 5 °C/min. The SBA-15 support samples modified with ammonium metavanadate, or vanadium(IV) oxide sulfate were labeled as V_M_/SBA-15 or V_S_/SBA-15, respectively.

### 2.4. Catalyst Characterization

The SBA-15 silica before and after modification with vanadium species was characterized by XRD measurements using a Bruker AXS D8 Advance diffractometer (Bruker, Karlsruhe, Germany) with Cu Kα radiation (λ = 0.154 nm). Data were collected in the low-angle range of 2θ = 0.6° to 8° and in the wide-angle range of 2θ = 6° to 60° with a resolution of 0.02° and 0.05°, respectively.

The textural properties of the catalysts were determined by N_2_ adsorption/desorption measurements using a Micromeritics ASAP 2020 instrument (Norcross, GA, USA). Prior to analysis, the sample was outgassed at 300 °C for 8 h. The specific surface areas were determined by the Brunauer–Emmett–Teller (BET) method, whereas the average pore diameter was determined using the Density Functional Theory (DFT) method.

UV–Vis spectra were recorded on a Varian-Cary 300 Scan UV–visible spectrophotometer with an integrated sphere CA-30I (Candela, Warszawa, Poland). Catalysts, first dried at 100 °C for 12 h, in the form of powders were placed into a cell equipped with a quartz window. The Kubelka–Munk function (F(R)) was applied to convert reflectance measurements into equivalent absorption spectra using the reflectance of SPECTRALON (Labsphere, Inc., North Sutton, NH, USA) as a reference.

XPS analyses were performed using an ultra-high vacuum photoelectron spectrometer based on the Phoibos 150 NAP analyzer (Specs, Berlin, Germany). The operating pressure in the chamber was close to 5 × 10^−9^ mbar. The materials examined were irradiated with monochromatic Al Kα radiation (1486.6 eV). Binding energies were referenced to the C 1s peak at 284.6 eV.

Pyridine adsorption followed by FTIR measurements was performed using a Bruker INVENIO S spectrometer with an in situ vacuum cell (Bruker, Poznan, Poland). Prior measurement, catalysts were formed into thin wafers and placed inside the cell. The cell with the sample was then outgassed at 350 °C for 2 h. After this step, pyridine was admitted at 150 °C. After saturation with pyridine, the solid was degassed at 150 °C, 200 °C, 250 °C, and 300 °C in vacuum for 30 min at each temperature. The spectrum without adsorbed pyridine (after sample outgassing at 350 °C for 2 h) was subtracted from all recorded spectra.

### 2.5. Catalytic Tests

#### 2.5.1. Catalytic Oxidative Desulphurization (CODS)

The catalytic oxidative desulphurization of dibenzothiophene was performed in a glass reactor using an EasyMax Workstation (Mettler-Toledo Inc., Columbus, OH, USA). The CODS was carried out using the following mixture: 500 ppm of dibenzothiophene (DBT) in dodecane (5 mL), 1 wt.% of a catalyst, and hydrogen peroxide (H_2_O_2_/DBT = 6). Prior to reaction, the catalyst was heated at 400 °C for 8 h to remove moisture. Then, the dried catalyst was put into the test tube of the EasyMax Workstation and DBT in dodecane was added. When the reaction mixture reached a temperature of 60 °C, hydrogen peroxide was admitted. The reaction was carried out for 120 min.

#### 2.5.2. Extractive Catalytic Oxidative Desulphurization (ECODS)

The extractive catalytic oxidative desulphurization of dibenzothiophene was performed in a glass reactor using the EasyMax Workstation. The ECODS was carried out using the following mixture: 500 ppm of dibenzothiophene (DBT) in dodecane (5 mL), acetonitrile (5 mL), 1 wt.% of a catalyst, and hydrogen peroxide (H_2_O_2_/DBT = 6). Prior to reaction, the catalyst was heated at 400 °C for 8 h to remove the moisture. Then, the dried catalyst was put into the test tube of the EasyMax Workstation and DBT in dodecane and acetonitrile were added. Ten minutes after the reaction mixture reached a temperature of 60 °C, hydrogen peroxide was admitted. The reaction was carried out for 120 min.

#### 2.5.3. Determination of Catalytic Activity

Differences in the DBT concentration were measured in the oil phase, from which the doses of reactants were taken in the appropriate time intervals. The conversion of DBT was determined using the following equation:

(1) $$\%\;\mathrm{Conversion}\;\mathrm{of}\;\mathrm{DBT}=\frac{{(C}_{0}-\;C)}{C_{0}}\;\text{×}\;100\%$$

where C_0_—concentration of DBT at the beginning of the reaction and C—concentration of DBT during the reaction progress.

A GC (Thermo Scientific, Waltham, MA, USA) equipped with 30 m DB-1 column and an FID detector was used to determine the DBT concentration during the CODS, EODS, and ECODS processes. Helium was applied as a carrier gas. The temperatures of the injector and the detector were set to 250 °C and 280 °C, respectively. The initial temperature of the column was set to 80 °C for 3 min and then increased to 300 °C (temperature ramp of 10 °C/min).

## 3. Results and Discussion

In this study, vanadium-containing catalysts were prepared by incipient wetness impregnation using two different sources of vanadium, i.e., ammonium metavanadate (NH_4_VO_3_) and vanadium(IV) oxide sulfate (VOSO_4_).

### 3.1. Characterization of the Catalysts

**Figure 1:**
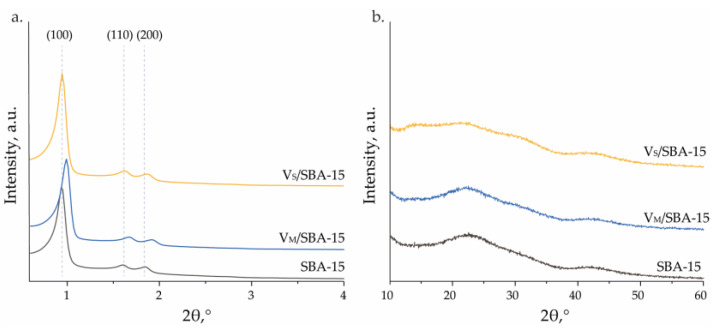
Small-angle (**a**) and wide-angle (**b**) XRD patterns of the obtained materials.

The successful synthesis of SBA-15 was confirmed by XRD measurements. The XRD pattern of the support is presented in Figure 1. The first and the most intense reflex corresponds to the (100) plane, and its position depends on the interspace distance between the walls of the hexagonal channels of the material. The two peaks corresponding to the (110) and (200) planes confirm that the above-mentioned channels show good long-range ordering. In general, it can be concluded that these three peaks detected in the XRD pattern confirm the typical hexagonal structure of the ordered SBA-15 (P6mm) material. The incorporation of vanadium species did not have a negative impact on the support structure or ordering of the pores, which is certified by the presence of reflexes assigned to the (100), (110), and (200) planes on the small-angle XRD patterns of V_M_/SBA-15 and V_S_/SBA-15. Only a slight shift in the reflexes towards higher values of 2 theta is observed for the V_M_/SBA-15 sample, which should be related to a small decrease in the interspace distance between the material walls. In the wide-angle XRD patterns of the prepared materials, no crystalline phase is detected (Figure 1). The patterns are very similar and typical of amorphous material. The lack of reflexes related to vanadium species suggests that they are also amorphous in nature or well dispersed and small enough to be not detected by XRD measurements.

**Figure 2:**
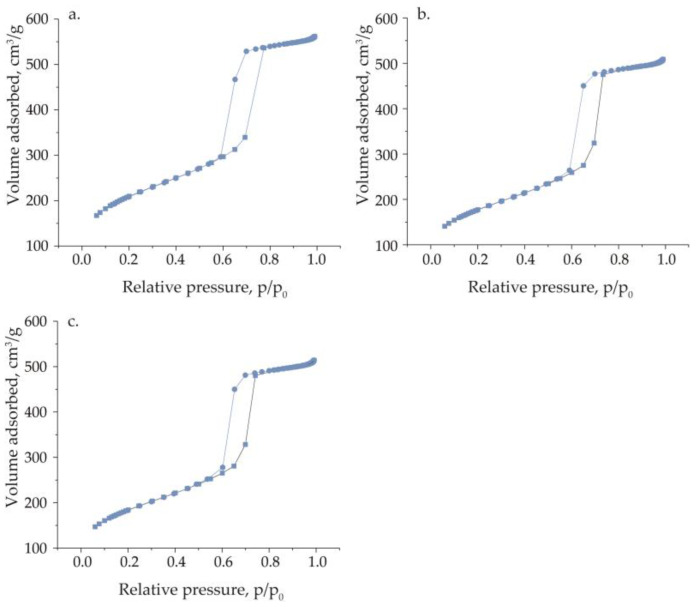
N_2_ adsorption/desorption isotherms of (**a**) SBA-15, (**b**) V_M_/SBA-15, and (**c**) V_S_/SBA-15.

More information related to the structure of the obtained materials can be deduced from low-temperature N_2_ adsorption/desorption measurements, the results of which are presented in Figure 2. The SBA-15 support shows isotherm characteristics of ordered mesoporous materials. This isotherm can be assigned to type IVa according to the IUPAC classification [22]. The characteristic feature of this isotherm is a step increase in the volume of adsorbed nitrogen in mesopores followed by a plateau for higher values of p/p_0_. Moreover, the desorption of nitrogen from mesopores needs a greater decrease in pressure, which results in the appearance of a hysteresis loop. The observed one can be assigned as type H1, typical of uniform-in-size mesopores. After the incorporation of vanadium species, regardless of the source of metal, the shape of the isotherm did not change. A decrease in adsorbed nitrogen volume is noted for samples with immobilized vanadium. This is in line with the results of XRD measurements, indicating that impregnation with vanadium sources followed by calcination did not affect the mesoporous structure of the support.

The textural parameters calculated from the low-temperature nitrogen adsorption data are presented in Table 1. SBA-15 has a relatively large specific surface area, which reaches 762 m^2^/g. The application of Pluronic P123 as a template for the synthesis of the mesoporous support also results in the microporosity of the final sample. The surface area of micropores reaches 176 m^2^/g for SBA-15. The specific surface area of the support decreases after incipient wetness impregnation with vanadium sources. A slightly greater decrease in this parameter is observed for the V_M_/SBA-15 material. Moreover, the pore diameter size distribution, presented in Table 1, indicates a decrease in pore diameter from 10.1 nm to 9.6 nm, which suggests the location of the modifier on the hexagonal walls of the SBA-15 support.

**Figure 3:**
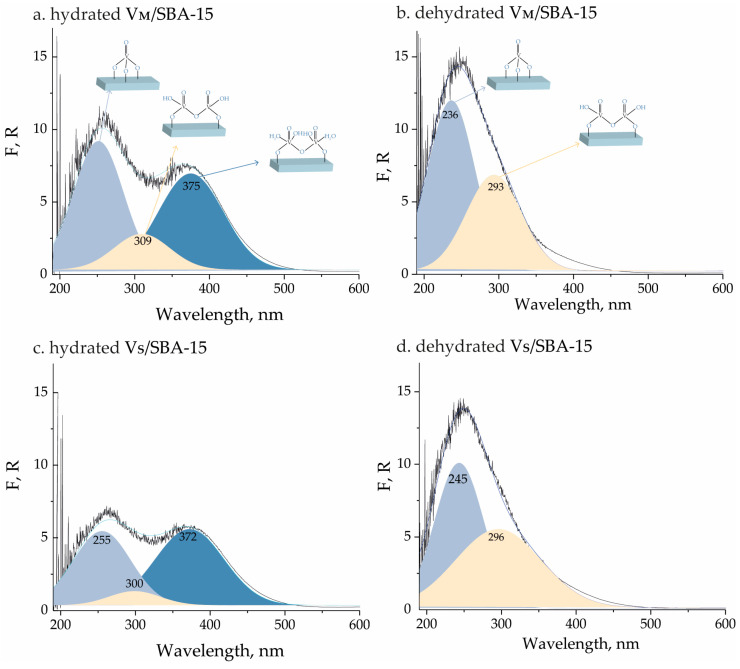
UV-Vis spectra of (**a**) hydrated V_M_/SBA-15, (**b**) dehydrated V_M_/SBA-15, (**c**) hydrated V_S_/SBA-15, and (**d**) dehydrated V_S_/SBA-15.

The coordination environment of vanadium species incorporated on the SBA-15 surface was investigated for hydrated and dehydrated samples using UV-Vis spectroscopy. The recorded spectra are presented in Figure 3. For the hydrated samples, three adsorption bands can be observed in the spectra. The first one located at ca. 255 nm is assigned to a low-energy transfer transition between oxygen and isolated vanadium species [23,24]. The presence of tetrahedrally coordinated oligomeric vanadium species was also confirmed by the adsorption band at ca. 300 nm [25]. The last band located at ca. 375 nm recorded for hydrated samples is attributed to pentagonal or pseudo-octahedral coordinated vanadium species that interact with moisture [26]. Also, the samples after thermal pretreatment at 250 °C for 2 h were subjected to UV-Vis measurements. The resulting spectra were different from those of the hydrated samples. The band located at ca. 375 nm was not observed, which indicated the removal of moisture from vanadium surroundings. Moreover, the domination of the band assigned to isolated vanadium species indicated good dispersion of vanadium species and a relatively low concentration of oligomeric species [27,28].

More detailed insight into the amount and oxidation state of vanadium species was achieved using X-ray photoelectron spectroscopy. The concentrations of vanadium species on the surface of the SBA-15 samples are presented in Table 1. The application of ammonium metavanadate (NH_4_VO_3_) as a source of vanadium species allowed for reaching a slightly higher concentration of vanadium relative to that when the source of vanadium was vanadium(IV) oxide sulfate, i.e., 1.42 wt.% vs. 1.12 wt.%. However, a more important difference was observed in the oxidation state of vanadium. The data related to binding energies and the percentage of surface vanadium species are collected in Table 2. In the spectrum of the V_M_/SBA-15 sample, two bands in the V 2p_3/2_ region were recorded. Based on the literature, the band at 516.9 eV can be assigned to the V^4+^ oxidation state [29,30,31], whereas the one located at 515.5 eV can be attributed to vanadium at the +3 oxidation state [30,32]. It can be concluded that in the V_M_/SBA-15 sample, both forms of V^4+^ and V^3+^ are present, with the former being dominant. For the V_S_/SBA-15 material, V^3+^ was not detected. The dominant form of vanadium was V^4+^; however, V^5+^ was also present on the catalyst surface. This clearly indicates the role of the vanadium precursor type used for modification on its oxidation state in the final material.

**Figure 4:**
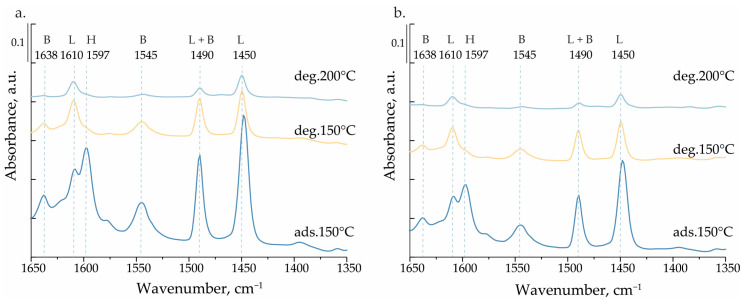
FT-IR spectra recorded for (**a**) V_M_/SBA-15 and (**b**) V_S_/SBA-15 after pyridine adsorption and degassing at different temperatures. H: hydrogen bonds, L: LAS, B: BAS.

The acidity of the materials obtained was examined by pyridine adsorption followed by FTIR measurements. The spectra after pyridine adsorption as well as after outgassing at different temperatures are presented in Figure 4. After adsorption of pyridine on the surface of V_M_/SBA-15 and V_S_/SBA-15, two bands at 1610 cm^−1^ and 1450 cm^−1^ appeared as a result of the interaction between the base molecule and the Lewis acid sites (LASs), most probably in the tetrahedral arrangement with V^4+^ coordinated to four oxygen species. In addition, the bands at 1638 cm^−1^ and 1545 cm^−1^ were also present in the spectra and provided evidence of the interaction between pyridine and Brønsted acid sites (BASs), which resulted in the formation of a pyridinium ion. The formation of BASs on vanadium-containing materials has been reported in the literature [33]. The intensity of the bands related to BASs significantly decreased after outgassing the sample in vacuum at 200 °C, which resulted in a decrease in the number of calculated BASs (Table 3). Also, the number of LASs decreased after outgassing in vacuum at 200 °C. Based on the data collected in Table 3, it can be concluded that the number of both LASs and BASs was greater for V_M_/SBA-15 than V_S_/SBA-15, which is in line with the concentration of surface vanadium species estimated by XPS and presented in Table 1.

### 3.2. Catalytic Oxidative Desulphurization of Dibenzothiophene

**Figure 5:**
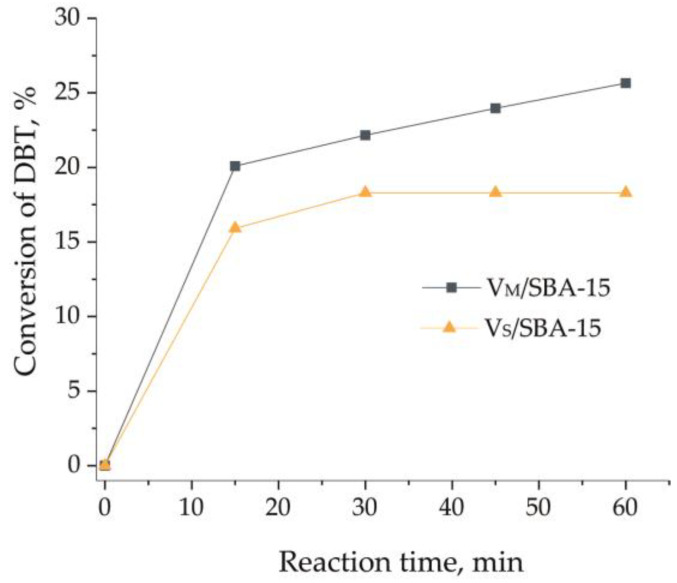
Catalytic activity of V_M_/SBA-15 and V_S_/ SBA-15 in CODS of DBT.

The activities of the materials obtained were tested in the catalytic oxidative desulfurization process using a mixture of dibenzothiophene (DBT, 500 ppm) in n-dodecane. The reaction was performed at 60 °C, and the molar ratio of H_2_O_2_/DBT was equal to 6. In the absence of a catalyst, the addition of hydrogen peroxide allowed for the oxidation of 4% of DBT after 1 h. The results obtained in the presence of catalysts are presented in Figure 5. In the presence of V_M_/SBA-15, the conversion of DBT reached ca. 20% after the first 15 min of the process and then slowly but systematically increased up to 25% at the end of the process. In contrast, for the V_S_/SBA-15 material, the conversion of DBT after the first 15 min reached 15%; however, no further oxidation of DBT was observed. A better catalytic activity of the V_M_/SBA-15 sample can be correlated with its higher acidity, estimated by pyridine adsorption (both LASs and BASs). Moreover, it is known from the literature that a lower oxidation state of a metal favors the efficiency in catalytic oxidative desulfurization [34]. It was documented that for the V_M_/SBA-15 sample, vanadium is present in a more reduced form than in V_S_/SBA-15. Therefore, one cannot rule out that not only the number of accessible surface vanadium species but also their form have a positive impact on the catalytic activity in CODS.

**Figure 6:**
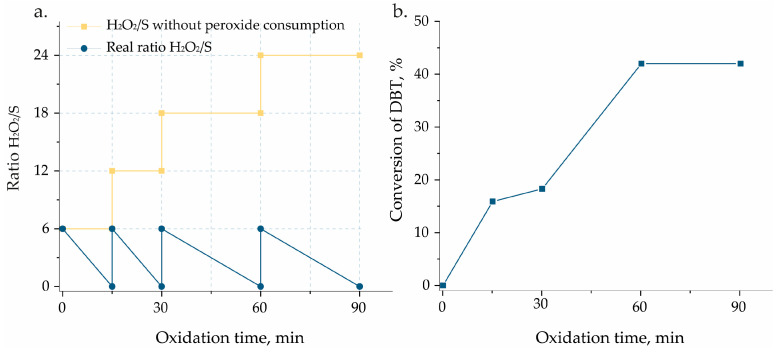
(**a**) Evaluation of the decomposition of H_2_O_2_ and (**b**) the catalytic performance of V_S_/SBA-15 with successive additions of H_2_O_2_ after 15, 30, and 60 min.

It is known from the literature that the presence of vanadium species is responsible for a substantial acceleration of H_2_O_2_ decomposition [35]. Therefore, one should consider the competition between the CODS and hydrogen peroxide decomposition processes. To examine this, the CODS process was performed in the presence of V_S_/SBA-15 catalyst; however, additional portions of hydrogen peroxide were added after each sampling, i.e., after 15, 30, and 60 min from the beginning of the reaction. Moreover, the concentration of H_2_O_2_ was measured in the reaction mixture by an iodometry-based titration technique. The results obtained are presented in Figure 6. It can be noted that after each sampling and before subsequent H_2_O_2_ addition, the concentration of hydrogen peroxide was equal to 0. It indicates that the majority of the oxidant was consumed not for the oxidation of DBT but was decomposed in the presence of vanadium species. Nevertheless, the systematic addition of hydrogen peroxide also makes it possible to increase the oxidation of DBT up to 40% at an accumulated H_2_O_2_/DBT molar ratio of 24.

### 3.3. Extractive Catalytic Oxidative Desulfurization of Dibenzothiophene

In an attempt to overcome problems with the fast decomposition of hydrogen peroxide in the presence of vanadium species, the materials were applied in a biphasic system, i.e., in extractive catalytic oxidative desulfurization (ECODS). The catalytic activities of the materials obtained were tested for a model reaction using a mixture of dibenzothiophene (DBT, 500 ppm) in dodecane, hydrogen peroxide as an oxidizing agent, and acetonitrile as a solvent (oil and solvent phases at the same volume ratios).

At first, efficiency in the extraction of DBT (EODS), i.e., the process carried out without hydrogen peroxide addition, was investigated. For this purpose, a mixture of DBT in dodecane was stirred with acetonitrile (MeCN) for 1 h. After the first 10 min of stirring, 53% of DBT was transferred to acetonitrile (Table 4). The concentration of DBT did not change during further stirring. The extraction of DBT in the presence of the SBA-15 support and vanadium-containing materials was a little bit higher and ranged from 55 to 59%. This small increase should be related to the adsorption of DBT on the surface of the mesoporous material.

**Figure 7:**
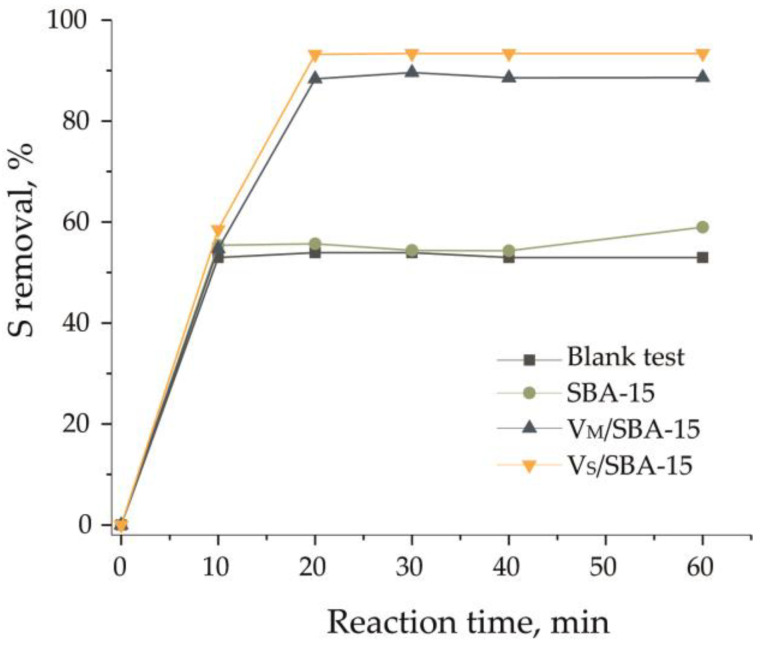
Desulfurization profiles of the ECODS process on the blank test (without catalyst), SBA-15, V_M_/SBA-15, and V_S_/SBA-15. Oxidation conditions: w(cat.) = 1.0%, H_2_O_2_/S = 6:1, 60 °C, and *v*(oil)/*v*(extractant) = 1:1.

The results of the ECODS process are presented in Figure 7. The same level of DBT removal was obtained for the blank test, i.e., carried out without catalyst addition, which was 53%. Also, the addition of the support did not change the result of the ECODS process. However, different results were obtained in the presence of vanadium-containing materials. The efficiency of DBT removal increased to 88% and 93% for V_M_/SBA-15 and V_S_/SBA-15, respectively. These results clearly indicate that the combination of oxidation and extraction processes can enhance the effectivity of vanadium-containing materials in DBT removal from the oil phase.

**Figure 8:**
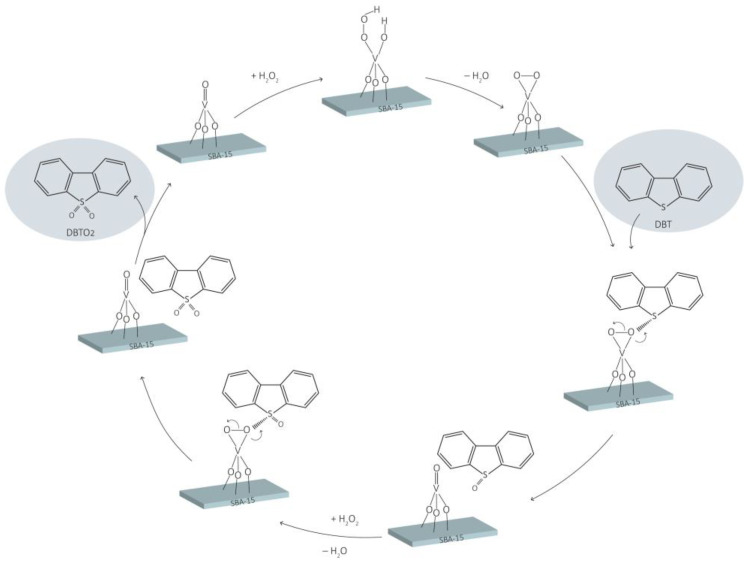
The proposed mechanism of DBT oxidation with H_2_O_2_ in the presence of vanadium-containing SBA-15.

The proposed mechanism of oxidative desulfurization with hydrogen peroxide in the presence of vanadium-containing silica SBA-15 is presented in Figure 8. According to the literature, in the first step, H_2_O_2_ is coordinated with the vanadium atom located on the silica surface. This leads to the formation of peroxometallic V-O-O-H species. Subsequently, the peroxovanadium complex V-O-O-V is formed by the elimination of the water molecule. Then, the electron pair located on the sulfur atom of DBT interacts with the peroxovanadium complex, leading to the formation of sulfoxide and regeneration of isolated V=O species. In the next step, the electron pair located on the sulfur atom of the formed sulfoxide interacts with another peroxovanadium complex. As a consequence, dibenzotiophene sulfone (DBTO_2_) is formed and isolated V=O species are regenerated [20,36,37,38].

**Figure 9:**
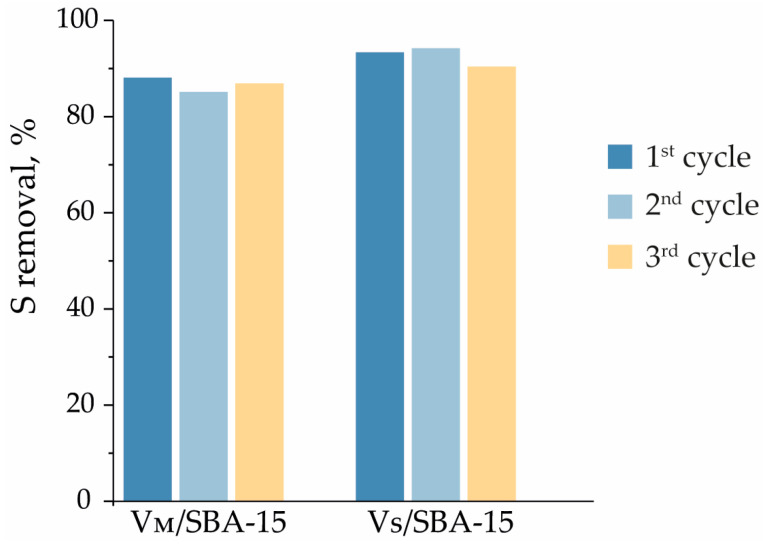
Regeneration of the catalyst. Oxidation condition: w(cat.) = 1.0%, H_2_O_2_/S = 6:1, 60 °C, 1000 rpm, 20 min, *v*(oil)/*v*(extractant), 1:1.

The common problem for processes carried out in the liquid phase is the leaching of the active phase of the catalyst to the reaction mixture, and, as a result, the catalytic activity would decrease in subsequent reaction cycles. This is especially important for the oxidation processes carried out in the presence of hydrogen peroxide, which strongly interacts with metal cations and may facilitate leaching. Therefore, vanadium-containing materials were also tested in the ECODS process in three cycles. The catalysts after the reaction were washed with 5 mL of acetonitrile, centrifugated, dried at 100 °C, and reused in a subsequent ECODS cycle. The results obtained are presented in Figure 9. It can be clearly seen that the results obtained for DBT removal are very similar for each reaction cycle.

A comparison of the catalytic activity in ECODS performed in the three-phase system, that is, the DBT solution in dodecane, hydrogen peroxide, and acetonitrile, under the vanadium-containing SBA-15 materials, presented in this work with samples described in the literature is shown in Table 5. It can be clearly seen that V_M_/SBA-15 and V_S_/SBA-15 have = high activity in DBT removal from the oil phase that is similar to the other listed catalysts.

## 4. Conclusions

Ammonium metavanadate and vanadium(IV) oxide sulfate were applied to prepare vanadium-containing SBA-15 materials via the incipient wetness impregnation method. The final materials were characterized and applied as catalysts for the catalytic oxidative desulfurization of dibenzothiophene (DBT) and also with simultaneous extraction. The kinds of surface vanadium species present in the support were determined by the type of vanadium salt used for the material preparation. The application of ammonium metavanadate allowed for obtaining samples containing a higher concentration of surface vanadium species and a higher number of acid sites of both Brønsted and Lewis acid types. At the same time, vanadium species were present at a lower oxidation state than in V_S_/SBA-15. This resulted in the superior catalytic activity of the V_M_/SBA-15 sample in the CODS process. The results evidenced that a fast decomposition of hydrogen peroxide did not allow for reaching a high efficiency of DBT removal, especially for V_S_/SBA-15. This problem was overcome in the process combined with simultaneous extraction. Both vanadium-containing materials showed high activities and stabilities in the ECODS process.

## Figures and Tables

**Table 1:** Textural properties of the catalysts and concentration of vanadium species.

Catalyst	BET, (m² g^−1^)	Pore Volume, (cm³ g^−1^)	S_Micro_, (m² g^−1^) ^a^	PSD_DFT_, (nm) ^b^	V Content, (wt.%) ^c^
SBA-15	762	0.83	176	10.1	-
V_M_/SBA-15	642	0.77	141	9.6	1.42
V_S_/SBA-15	667	0.77	157	9.6	1.12

^a^ S_Micro_—surface area of micropores according to the t-plot method; ^b^ PSD_DFT_—pore size distribution (PSD) calculated by the DFT method; ^c^ V—vanadium content calculated by XPS analysis.

**Table 2:** XPS BE values of vanadium species and their concentrations.

BE, eV	V_M_/SBA-15	V_S_/SBA-15
V^5+^	-	517.7 (10.8%)
V^4+^	516.9 (58.4%)	516.6 (89.2%)
V^3+^	515.0 (41.6%)	-

**Table 3:** The number of Lewis (LASs) and Brønsted (BASs) acidic centers.

	LAS, μmol/g		BAS, μmol/g	
	150 °C	200 °C	150 °C	200 °C
V_M_/SBA-15	49	21	35	6
V_S_/SBA-15	40	14	26	4

**Table 4:** Removal of dibenzothiophene (DBT) from the oil phase in different desulfurization systems.

Catalyst	CODS Catalyst + H_2_O_2_	EODS Catalysts + MeCN	ECODS Catalyst + MeCN + H_2_O_2_
Blank	3	53	53
SBA-15	0	55	55
V_M_/SBA-15	26	55	88
V_S_/SBA-15	18	59	90

**Table 5:** The comparison of activity in ECODS of vanadium-containing mesoporous silicas SBA-15.

Catalyst	V Source	H_2_O_2_/S	Time, min	Temp., °C	S Removal, %	Ref.
VOx-Ga-SBA-15 (4 wt.% Ga, 6 wt.% V)	VCl_3_	5	15	60	98	[18]
VOx-Ga-SBA-15 (0 wt.% Ga, 6 wt.% V)	VCl_3_	5	15	60	65	[18]
10% V_2_O_5_/SBA-15	NH_4_VO_3_	10	20	60	75	[20]
10% V_2_O_5_/Zr-SBA-15	NH_4_VO_3_	10	20	60	90	[20]
VOx-Ga-SBA-15 (1/30)	VCl_3_	6	15	60	99	[39]
VOx-SBA-15 (1/30)	VCl_3_	6	15	60	99	[39]
V-SBA-15 (1/30)	VCl_3_	6	15	60	85	[39]
V_M_/SBA-15	NH_4_VO_3_	6	20	60	88	This work
V_S_/SBA-15	VOSO_4_	6	20	60	93	This work

## Data Availability

The data presented in this study are available on request from the corresponding author via e-mail.
